# Characteristics of the Tumor Microenvironment That Influence Immune Cell Functions: Hypoxia, Oxidative Stress, Metabolic Alterations

**DOI:** 10.3390/cancers12123802

**Published:** 2020-12-17

**Authors:** Ryan C. Augustin, Greg M. Delgoffe, Yana G. Najjar

**Affiliations:** 1 Department of Medicine, University of Pittsburgh, Pittsburgh, PA 15260, USA; 2 Department of Immunology, University of Pittsburgh, Pittsburgh, PA 15260, USA; delgoffeg@upmc.edu; 3 Division of Hematology/Oncology, Department of Medicine, University of Pittsburgh, Pittsburgh, PA 15260, USA; najjaryg@upmc.edu

**Keywords:** immunometabolism, immunotherapy, cellular energetics

## Abstract

**Simple Summary:**

For decades nearly all cancer patients were treated with cytotoxic chemotherapy, killing any rapidly dividing cell in the body. More recently, researchers have been studying the immune system’s response to cancer and have developed a novel class of drugs that stimulate the body’s own response to tumors. This class of immunotherapy drugs primarily involve T-cells, immune cells that target and destroy cancer cells. While these drugs can lead to remarkable and sustained response, most patients do not respond. Understanding the resistance mechanisms in non-responding tumors is now an active area of investigation. In this review, we explore a host of factors in the tumor microenvironment, the cellular and molecular space within tumor tissue, to identify possible culprits of immunotherapy resistance. Specifically, the downstream effects of low oxygen and metabolic byproducts in the tumor microenvironment have been associated with immune cell dysfunction. Importantly, targeting these pathways may offer promising therapies to improve the response to current immunotherapy.

**Abstract:**

Immunotherapy (IMT) is now a core component of cancer treatment, however, many patients do not respond to these novel therapies. Investigating the resistance mechanisms behind this differential response is now a critical area of research. Immune-based therapies, particularly immune checkpoint inhibitors (ICI), rely on a robust infiltration of T-cells into the tumor microenvironment (TME) for an effective response. While early efforts relied on quantifying tumor infiltrating lymphocytes (TIL) in the TME, characterizing the functional quality and degree of TIL exhaustion correlates more strongly with ICI response. Even with sufficient TME infiltration, immune cells face a harsh metabolic environment that can significantly impair effector function. These tumor-mediated metabolic perturbations include hypoxia, oxidative stress, and metabolites of cellular energetics. Primarily through HIF-1-dependent processes, hypoxia invokes an immunosuppressive phenotype via altered molecular markers, immune cell trafficking, and angiogenesis. Additionally, oxidative stress can promote lipid peroxidation, ER stress, and Treg dysfunction, all associated with immune dysregulation. Finally, the metabolic byproducts of lipids, amino acids, glucose, and cellular energetics are associated with immunosuppression and ICI resistance. This review will explore these biochemical pathways linked to immune cell dysfunction in the TME and highlight potential adjunctive therapies to be used alongside current IMT.

## 1. Introduction

Metabolism serves as a unifying theme to the complex set of cells and cellular processes of the heterogeneous tumor microenvironment (TME). Indeed, all cells require fuel to perform their diverse functions, and the metabolic by-products of cellular energetics are a key component of the TME [1]. As the field of immunotherapy continues to grow, our understanding of this metabolic meshwork has become a critical factor in optimizing immune cell function [2]. Although immune checkpoint inhibitors (ICI) and other novel immune-based therapies have revolutionized the current treatment paradigm of many cancers, only a minority of patients show sustained response [3]. Thus the burgeoning field of immunometabolism is now being harnessed to highlight the interconnecting elements of the TME that may lead to a dysfunctional immune state [2].

The widespread metabolic dysregulation found in the TME creates numerous hurdles but also provides key insight into more effective combinatorial approaches with ICI. Given the plasticity and dynamic nature of the TME, reversion to an immune-responsive state will require a more detailed schema into the timing and precise nature of combination therapies [4,5]. Furthermore, the signaling pathways of immune suppressor cells (e.g., Treg, MDSC) can be hijacked by metabolic mediators and further disrupt cytotoxic T cell activity in the setting of ICI [6]. Based on many more examples that will be described below, it is clear that the harsh metabolic milieu of the TME requires reversal with novel therapies to unleash the full benefit of ICI and other immune-based therapies, and extend the life saving benefits of immunotherapy to a higher number of patients.

We have chosen to categorize the primary immune components of the TME based on the most evidence-based pre-clinical models, and the potential implications for clinical translation. These three categories include: hypoxia, oxidative stress, and metabolic alterations. We will highlight several pioneering discoveries from the last decade that will have broad implications for the field of immunotherapy in the coming years.

## 2. Hypoxia

Hypoxia is perhaps the most well-studied perturbation found in the TME. The translational ramifications of tumor hypoxia were described in detail over 60 years ago, when it was discovered that hypoxic tumor cells became resistant to radiation therapy [7]. Over the ensuing years, hypoxia became a central theme in the pathophysiology of tumor growth, especially as it related to the evasion of cytotoxic therapies [8]. The original “Hallmarks of Cancer,” though focused on genetic perturbations leading to unrestrained proliferation, did highlight hypoxia as a key signal in promoting VEGF-mediated angiogenesis [9]. In the revolutionary era of immunotherapy, hypoxia has been recognized as a fundamental link between the emerging hallmarks of “tumor-promoting inflammation” and “deregulated cellular energetics” [10]. As the resistance to ICI—and how to overcome it—has become a primary focus in oncology, hypoxia and its relation to several components of the TME is now a key target for the next phase of immune-based therapies. As we explore these novel therapies, it is first essential to understand the pathophysiology of hypoxia in the TME: in this section we will review hypoxia as it relates to (a) molecular markers, (b) immune cell dysfunction, (c) tumor and stromal cell interaction, and (d) angiogenesis in the context of a tumor-induced immunosuppressive phenotype.

Although the implications of tumor hypoxia are well-documented, the exact etiology behind low oxygen levels in the TME are perhaps less well understood. The unregulated and explosive growth of cancer leads to a chaotic conglomeration of tumor and stromal cells with a dysfunctional vascular supply and poor access to nutrients [11]. Additionally, as will be described below, the metabolism and energy production of aggressive tumors can shift towards a highly oxidative state leading to hypoxic conditions for the remainder of the TME [12].

### 2.1. Molecular Markers

The hypoxia inducible factor (HIF) proteins are a set of transcription factors that are responsible for many of the phenotypic characteristics of the hypoxic TME. HIF-1α forms a heterodimer with HIF-1β; this complex binds to the hypoxia-responsive element (HRE) to induce expression of an assortment of genes used for normal tissue adaptation to low oxygen levels but also the aforementioned immunosuppressive phenotype of the TME [13]. As previously described by Noman et al., a variety of immune-related molecular markers are upregulated in response to low oxygen levels in the TME [14]. The first, and perhaps most relevant to current ICI, involves PD-L1, a ligand found on the surface of antigen-presenting cells and many tumor cells that binds to the PD-1 receptor on activated T cells [15]. HIF-1α, activated under hypoxic conditions, has been shown to directly upregulate expression of PD-L1, a process that inhibits further T-cell activation and signaling upon contact with PD-1 [16]. Another checkpoint found on myeloid derived suppressor cells (MDSC), V-Domain Ig Suppressor of T-Cell Activation (VISTA) was also shown to be upregulated in hypoxic regions of a colon cancer (CT26) model via HIF-1α [17]. Finally, the immune checkpoint CD47 has also been shown to be more highly expressed under hypoxic conditions. This protein found on the surface of tumor cells binds to macrophage and dendritic cells preventing phagocytosis, a defense against the innate immune system. While CD47 has been a more well-established biomarker and negative predictor in hematologic malignancies, recent studies with breast and pancreatic cancer have also revealed an association between CD47 expression with HIF-1α [18,19]. Finally, a non-classical MHC-I variant, HLA-G, has been shown to have several HREs in its promoter region and induced expression under hypoxic conditions. HLA-G presents peptide fragments on the surface of tumor cells, binds to several different proteins expressed by both lymphocytes and innate immune cells, and has well-documented associations with immune suppression and poor prognosis in a variety of tumor types [20].

### 2.2. Immune Cells

There are a variety of innate and adaptive immune cells that functionally mediate the immunosuppressive phenotype of the TME. The work of Chouaib et al. has highlighted many of the immune cells affected by the hypoxic conditions in cancer—namely, tumor-associated macrophages (TAM), MDSCs, regulatory T cells (Treg), and of course, cytotoxic T cells (CTL) [6]. A variety of TAM subtypes exist in the TME, with the M2 type exhibiting immunosuppressive characteristics. Data from hepatocellular carcinoma (HCC) models have shown significant correlation between HIF-1α expression and an increased proportion of M2 macrophages [21]. TAMs have also been associated with increased matrix metalloproteinase-7 (MMP-7) expression in hypoxic regions of the TME; MMP-7 inhibits tumor cell lysis via cleavage of the Fas ligand, a transmembrane protein involved in apoptosis [22,23]. Another cell line from the myeloid lineage, MDSCs, have even more data tied to hypoxia and immunosuppression. MDSCs were first called “natural suppressor” cells upon recognition of their potent inhibition of proinflammatory cytokines and both CD8+ and CD4+ T-cell activation [24]. The discovery of increased peripheral circulation of MDSC in tumor models led to their further characterization as detrimental “immune suppressor” cells in the TME [25,26,27]. Hypoxic conditions have been shown to further stimulate the immunosuppressive function of these cells; MDSC PD-L1 expression is also strongly upregulated in response to HIF-1α [28]. MDSCs in hypoxic tumors suppress both antigen-specific and antigen non-specific T cells, contributing to a more depleted TIL state [29]. HIF-1α has also been shown to increase differentiation of MDSCs into TAMs, with functional ramifications [30]. Moving to the lymphoid lineage, Tregs are likely the most recognized immunosuppressive cells found in the TME. Derived from CD4+ T cells, Tregs inhibit innate and adaptive immune function via cytokine signaling (TGF-β, IL-10) and checkpoint interactions (CTLA-4, LAG3) among other mechanisms [31]. The expression of FOXP3, a differentiating biomarker of CD4+ to Treg morphology, is directly upregulated via HIF-1α [32]. Various chemokines (CCL-17, CCL-28, TGF-β1 itself) secreted by hypoxic tumors also attract Tregs to the TME [33]. As a functional example, Hasmim et al. sought to inhibit the chemoattractant TGF- β1 by blocking one of its transcription factors, NANOG; this led to a significant decrease in Treg infiltration in a B16-F10 melanoma tumor bed [34]. Finally, in describing the effects of hypoxia on CD8+ T cells, the picture is less clear. As described by Vuillefroy de Silly et al., a dichotomy of both in vitro and in vivo CD8+ T cells under a varied range of normoxic and hypoxic conditions has made experimental results difficult to summarize [35]. On one hand, T-cell expansion is diminished under hypoxic conditions, possibly due to disrupted T-cell receptor (TCR) signaling. On the other hand, no compelling evidence has linked HIF-1α expression with decreased CTL function in the TME. Furthermore, the effects of hypoxia on CTL cytolytic capabilities have been mixed [35], although our work with antigen-specific T cells revealed lower IFN-γ levels and depressed cytolytic activity under acutely hypoxic conditions [36]. While more research is needed to further characterize the effects of hypoxia on CTL function, these crucial cells have clearly evolved to adapt to a wide range of harsh environments. In general, the detrimental effects of hypoxia are spread amongst most types of immune cells in the TME.

### 2.3. Tumor and Stromal Cells

While immune cells are of significant interest in the era of immunotherapy (IMT), tumor cells themselves along with the tumor-stromal interaction create a climate of immune resistance that is enhanced by hypoxia. Autophagy is an oft-cited resistance mechanism of tumor cells in which altered endosomes degrade cytolytic proteins (e.g., granzyme B, perforin) released by NK and CTLs [37]. Recent work has demonstrated that hypoxia, via a HIF-1α- and pSTAT3-dependent mechanism, upregulates this process leading to selective degradation of granzyme B and depressed cytolysis [38]. Cancer-associated fibroblasts (CAF) are one of the main inducers of extracellular matrix (ECM) remodeling, a deleterious process that enhances tumor growth, immune resistance, and migration [39]. Under hypoxic conditions, CAFs have been shown to induce tumor progression in a pancreatic cancer model via arginase II secretion, a mechanism that leads to T cell anergy and a depressed immune state [40]. Finally, the unique metabolic interaction between CAFs and tumor cells can lead to a “corrupted” nutritional state in the TME that further disrupts immune cell function [41]. Stromal cells populating the most hypoxic conditions of the tumor will rely on glycolysis, produce large amounts of lactate/pyruvate intermediates, and “feed” neighboring tumor cells that can participate in the more valuable oxidative process to stimulate hyperproliferation [42]. In this “reverse Warburg effect” model, oxygen is a rare and important cofactor in the TME with repercussions on immune cell function, as previously described [42]. Further ramifications of this model and other metabolic alterations will be described in the upcoming sections.

### 2.4. Angiogenesis

Another element interconnecting hypoxia and the immune state is the blood vessels that carry oxygen to—and waste products out of—the TME. The role of angiogenesis contributing to a pathological TME has been studied for nearly fifty years; tortuous, chaotic, leaky vessels lead to elevated interstitial pressure, disorganized oxygen delivery, and inefficient nutrient transfer [43]. Indeed, the critical role of angiogenesis in cancer biology has long been appreciated, and anti-angiogenic therapies have been successfully used in several cancer types, including renal cell carcinoma and colorectal cancer. While tumor cells are constantly mutating and adapting to their harsh climate, surrounding “normal” cells and tumor infiltrating immune cells struggle to compete. Tumor angiogenesis is primarily mediated through a set of VEGF growth factors and their family of receptors (VEGFR); angiopoietin and various adhesion molecules are also involved [44]. Disrupting angiogenesis via VEGF inhibitors initially held promise as a means of nutrient and oxygen blockade to tumor cells, however, most do not respond to monotherapy [45]. Further disruption of already dysfunctional vessels has been shown to increase intratumoral hypoxia; a vicious cycle that upregulates HIF-1α, exacerbating many of the resistant mechanisms already described [46]. Indeed, while anti-angiogenic therapy was first thought to “starve” the tumor by cutting off blood supply, it is now appreciated that oxygen tension and immune infiltration are critical to maintain antitumor activity. This is especially important when considering partnering an anti-angiogenic with an immune-based therapy. More recent investigations have sought to “normalize” the vasculature of the TME via transient VEGF blockade to stabilize the endothelium, increase oxygen and drug delivery, and reduce vascular permeability with associated metastatic potential [47]. As explained by Ramjiawan et al., prudent timing of both anti-angiogenic and ICI therapies could reprogram and stabilize the TME and allow infiltrating lymphocytes to properly function before resistant mechanisms (many secondary to hypoxia) develop [43]. As of 2020, there are now several early phase clinical trials looking into various combinations of VEGF(R) + PD1/L1 blockade in colorectal, hepatocellular, prostate, melanoma, and other cancers [48,49,50,51,52,53]. Additionally, in advanced renal cell carcinoma (RCC) where VEGF inhibition has already been standard therapy, a phase III trial (KEYNOTE-426) showed improved outcomes with combination PD1 + VEGF therapy as compared to VEGF inhibition alone [54].

### 2.5. Targeting Hypoxia

When considering drug therapy related to hypoxia and the TME, it is important to first recognize the heterogenous state of intratumoral immune infiltration and the limitations of ICI monotherapy. Because of the dichotomous results seen in early clinical trials with ICI, a spectrum of tumor histologic subtypes were explored and characterized to better explain these differential outcomes [55]. Tumors with favorable ICI response, high density of IFN-γ producing CTLs, high tumor mutation burden or microsatellite instability (i.e., neoantigen production) can be categorized as “inflamed” TMEs with pre-existing immunity. The other end of the spectrum includes “noninflamed” TMEs with diminished CTL infiltration, low PD-L1 expression, and low mutational burden (i.e., immunologically ignorant) [55]. Tumors between these extremes are characterized by moderate CTL infiltration and neoantigen production but with hampered immune activity secondary to dysfunctional angiogenesis, hypoxia, and suppressive immune cells, cytokines, and metabolic cofactors. This “middle” group of tumors has the greatest potential to benefit from combinatorial therapy with ICI, with the aim of reversing some of the detrimental immunosuppressive effects of hypoxia (among others) and restoring a more functional “inflamed” TME [56,57].

Given the intense focus on hypoxia, one would surmise that blocking the primary transcriptional regulator (i.e., HIF-1α) would be a key opportunity to correct the immunosuppressive climate of the TME. However, given the ubiquity of this transcription factor and its role in controlling multiple cellular processes, the lack of specificity and risk of toxicity have mired the investigations of direct HIF-1α inhibitors [14]. Thus, indirect inhibitors of HIF-1α and blockade of downstream effector molecules have become a more pertinent area of research [58]. A host of agents have been discovered with incidental HIF-1α modulation; these include antisense oligonucleotides, the translational inhibitor and microtubule-targeting metabolite 2-methoxyestradiol (2ME2), heat shock protein inhibitors, histone deacetylase inhibitors, and even topotecan (a topoisomerase I inhibitor) among others [59]. Many of these drugs are under phase I clinical trial investigation in combination with various chemotherapies, although there are limited data on ICI combination. Over the last twenty years, a novel approach involving oxidation-reduction (redox) reactions has led to the development of hypoxia pro-drugs [60]. These prodrugs are activated via redox under extremely hypoxic conditions (<0.5% O_2_) with most active drugs leading to cytotoxic activity via DNA cross-linking [61]. After early promise [62], a phase III clinical trial (MAESTRO) is now underway, studying the effects of the hypoxia prodrug evofosfamide with gemcitabine in patients with pancreatic adenocarcinoma [63]. This same prodrug has also been studied pre-clinically in combination with anti-CTLA4 and anti-PD1 antibodies in a murine prostate cancer model [64]. Dual therapy led to a significant reduction in tumor growth with an elevated CTL to MDSC ratio as compared to either evofosfamide or ICI alone [64]. Finally, antagonists of two hypoxia-driven elements, CAIX and A2AR, are also under investigation with the goal of combating immunosuppression in the TME. The carbonic anhydrase enzyme, CAIX, is upregulated via HIF-1α and a significant prognostic marker in RCC; a preclinical study of anti-CAIX antibodies (girentuximab) was shown to stimulate both innate and adaptive immune-mediated killing of tumor cells [65]. The phase III ARISER clinical trial showed no significant survival benefit between monotherapy girentuximab and placebo in the adjuvant setting [66], though this has yet to be evaluated in the metastatic setting. The adenosine A2A receptor (A2AR) has been associated with advanced pathological grade along with HIF-1α expression in head and neck squamous cell carcinoma (HNSCC) [67]. An A2AR antagonist, SCH58261, was shown to not only decrease tumor growth but also increase the ratio of CTL to Treg cells in a murine HNSCC model [67]. Based on these preclinical data, multiple phase I clinical trials evaluating A2AR blockade plus anti-PD(L)1 are underway in advanced solid tumors [68,69]. In general, modulators of HIF-1 and its downstream elements have shown strong potential to reverse immunosuppressive factors in the TME; future clinical trials should be aimed at combinatory methods with ICI and other immune-based therapies (see Table 1) [70].

## 3. Oxidative Stress

The role of oxygen is two-fold in regards to TME immunosuppression. While hypoxia may be one of the most studied elements, evidence also points to oxidative stress as an important byproduct leading to a dysregulated immune state [71]. Originally studied in the field of chronic inflammation secondary to autoimmune and infectious diseases, lipid peroxidation has more recently been linked to several carcinogenic effects. Reactive oxygen species (ROS) and free radicals can preferentially oxidize fatty acids through lipoxidation, a process that leads to reactive aldehydes with numerous downstream consequences [72]. As detailed by Martin-Sierra et al., a specific aldehyde, HNE, has demonstrated both anti-tumorigenic effects via NFkB inhibition as well as immunosuppression depending on the relative concentration and specific HNE adduction [73]. One of these protein-HNE adducts has been shown to inhibit the phagocytic properties of innate cells in the TME [74]. Other HNE reactions have been associated with altered cytokine signaling in macrophages [73]. Thus, while more work is needed to summarize the effects of lipoxidation on downstream immune functions, harnessing this important redox process could hold promising therapeutic options in the future.

The endoplasmic reticulum (ER), the protein-folding workhorse of the cell, is also significantly affected by oxidative stress; this state of ER stress has been linked to various pro-tumorigenic effects, including immunosuppression [75]. A cervical cancer murine model was found to have increased tumor-infiltrating MDSCs with enhanced immunosuppressive capacity upon administration of thapsigargin, a specific ER stress inducer [76]. Furthermore, XBP1, a multimodal transcription factor associated with MHC II regulation along with the ER stress response was found to impede tumor immunogenic cell death in a colorectal cancer model [77]. While these examples have been specific to the tumor cells themselves, immune cells are also affected by ER stress. Early in vitro trials showed that ER stress led to dysfunctional antigen-presenting dendritic cells and an increase in immunosuppressive signaling molecules (e.g., arginase) [78]. Additionally, Cubillos-Ruiz et al. analyzed tumor-infiltrating dendritic cells within ovarian tumors—elevated ROS levels led to increased lipid peroxidation, HNE generation, and resultant ER stress [79]. Given these findings, several therapeutic options have been proposed: antioxidants to control ROS production, lipid uptake blockade to prevent peroxidation and HNE formation, and conditional knockout of *XBP1* in specific immune cells [75]. Per the latter, *XBP1* deletion in dendritic cells was found to stimulate CTL activity and delay tumor progression in multiple ovarian tumor models [79]. Again, further validation in both preclinical and clinical settings are warranted, but the effects of ER stress on TME immune activity may provide a window of therapeutic opportunities. Furthermore, lipid peroxidation and HNE could serve as important biomarkers for an immunosuppressed phenotype [75,80].

The role of Tregs in the TME has been thoroughly investigated, but more recent evidence points to a novel mechanism of immunosuppression related to oxidative stress. Maj et al. studied the role of oxidative stress-induced apoptotic Treg cells in the TME [81]. Surprisingly, the apoptotic cells were shown to have superior immunosuppressive qualities as compared to the live counterparts. Specifically, both the functional secretion and expression levels of CTL IL-2 and IFN-γ were significantly lower when exposed to apoptotic Treg cells. While immunoblotting revealed high levels of PD-L1, CTLA-4, CD39, and CD73 in the apoptotic cells, PD-L1 and CTLA-4 blockade did not mitigate CTL suppression. Thus, supernatant analysis was performed and showed that novel, small molecules were responsible for this increased suppression rather than well-characterized cytokine proteins (e.g., TGF-β, IL-35, IL-10). Further assays revealed that adenosine (via its A2A receptor, a known immunosuppressive mediator) was one of these culprit molecular factors [67]. Finally, researchers compared the results of oxidative stress on Treg versus CTL cells (via H_2_O_2_ administration); expression of the NRF2 antioxidant gene family was significantly lower in the Treg cells and likely the responsible factor for the high rate of apoptosis [81]. This experiment not only characterizes a unique functional mechanism of Treg immunosuppression, it also highlights oxidative stress as another key mediator of ICI resistance.

## 4. Metabolic Alterations

Thus far, we have detailed the important effects of both hypoxia and oxidative stress on the immune state of the TME. However, much of the basis for these and other immunosuppressive factors derives from deregulated energy metabolism, an “emerging hallmark” of cancer as described in Weinberg’s 2011 landmark paper [10]. This overarching metabolic category is flanked by two additional hallmarks, “avoiding immune destruction” and “tumor-promoting inflammation,” a collective foreshadowing into the pioneering research of immunometabolism.

### 4.1. Glycolysis and Oxidative Phosphorylation

The oft-touted Warburg hypothesis has shaped the metabolic vernacular of carcinogenesis for the last century [82]; however, this primary focus on aerobic glycolysis is a limited and archaic vantage point for understanding cellular energetics in the TME. An increase in glycolytic flux has certainly been demonstrated in many cancers, although glycolysis alone is not sufficient for energy production or tumor progression [83]. Indeed, several activating mutations within the PI3K-AKT-mTOR pathway are linked to higher glycolytic rates, yet the competitive advantage is likely tied to enhanced production of glycolytic intermediates rather than ATP generation [84]. These glycolytic intermediates are shunted into various anabolic pathways for nucleotide, protein, and lipid synthesis as well as oxidative metabolism for enhanced ATP production in less hypoxic regions of the TME. This symbiotic transfer of metabolites has been neatly described as the “reverse Warburg” effect [85], however, the heterogenous nature of each cancer precludes an assumption of any one consistent model. Regardless, increasing evidence points to an equal if not more critical dependence on mitochondrial metabolism as compared to glycolysis in regards to tumor progression and immunosuppression [86]. For example, in addition to studying the oncogenic PI3K/AKT pathway, loss of the p53 tumor suppressor has also been shown to increase the flux of glycolytic intermediates [87]. However, mitochondrial inhibition (via metformin) of p53 (−/−) colon cancer xenografts selectively leads to significant growth suppression, demonstrating that increased glycolysis alone does not explain the unchecked pattern of tumor proliferation [88].

To further characterize the role of glycolytic and oxidative tumor metabolism, oxygen as a critical metabolite, and the collective effects on immunotherapy, we developed a series of experiments modelling TIL function in the TME. Scharping et al. analyzed the rates of glycolysis and oxidative phosphorylation (OxPhos) using a Seahorse Bioanalyzer, which measures extracellular acidification rate (ECAR) and oxygen consumption rate (OCR), respectively [36]. Both melanoma and colon cancer tumor cells were measured, and although there was no difference in ECAR, the melanoma model had significantly higher OCR. Furthermore, TILs derived from the melanoma tumor demonstrated increased levels of hypoxia along with lower IFN-γ secretion and decreased cytolytic activity. Finally, complex I inhibition via metformin reversed both hypoxia and TIL dysfunction in the previously high OCR melanoma model. Not only are these results consistent with the previous discussion of hypoxia and its detrimental effects on TIL function, but it links these effects to a core metabolic driver—oxidative metabolism. In further work, we developed murine melanoma models with either *Glut1* (glycolytic) or *Ndufs4* (OxPhos) knockdown tumors. OxPhos-deficient tumors showed less hypoxia and more functional TIL activity as compared to control and *Glut1* knockdown tumor samples [89]. Additionally, only the OxPhos-deficient tumors showed response to anti-PD1 treatment, highlighting the impact of OxPhos-mediated hypoxia on TIL function and ICI efficacy. As a clinical correlate, tumors from 19 patients with melanoma were biopsied; higher OxPhos was significantly associated with decreased TIL function and higher TIL exhaustion, non-response to anti-PD1 therapy, and also decreased progression free and overall survival [89]. Based on these preclinical data, the phase I clinical trial investigating pembrolizumab plus metformin in advanced melanoma is now underway (NCT 03311308) [90]. An additional analysis of melanoma brain metastases (MBM) revealed a significant increase in OxPhos levels, both via genetic expression and metabolite profiling [91]. These highly oxidative tumors correlated with increased immunosuppression and reduced survival in murine MBM models; reversal of these effects was achieved using a novel OxPhos inhibitor, IACS-010795, a drug currently being investigated in multiple phase I clinical trials [92,93]. Understanding the impact of tumor cell metabolism on response to ICI has broad clinical implications, and will need to be evaluated across various tumor types. These results suggest that oxidative metabolism may serve as a predictive marker for response to ICI and other TIL-based therapies.

### 4.2. Amino Acids

While the interplay of glycolysis and OxPhos have distinct implications for the immune state of the TME, a number of other metabolic alterations also affect CTL function. For example, amino acids play a large role in tumor-mediated anabolic production, and competition between cancer cells and T cells can be a prime source of immunosuppression [94]. Yang et al. describe a “glutamine loop” in an ovarian cancer model in which CAFs convert the metabolic byproducts glutamate and lactate to glutamine; amino acid transporters upregulated in tumor cells rapidly import glutamine and re-export glutamate and lactate into the extracellular space to repeat the process [95]. While all amino acids are critical for cellular proliferation and protein biosynthesis, glutamine in particular has been shown to correlate with T-cell activation; in conjunction, decreased glutamine concentration has been tied to dysfunctional TIL activity [96]. In similar competitive fashion, arginine is preferentially imported by proliferating tumor cells; increased intracellular concentration has also been tied to elevated TAM infiltration in the TME, an immunosuppressive macrophage as described above [97]. As with glutamine, arginine has also been shown to be a necessary cofactor for T cell survival and anti-tumor activity [98]. Finally, a catabolic enzyme within the tryptophan pathway, indoleamine 2,3-dioxygenase-1 (IDO1), has gained recent interest due to its significant immunosuppressive effects. IDO1 was first studied due to associations between expression level and poor prognosis in a variety of cancers [99]. Subsequently, tryptophan catabolites (within the Trp-Kyn-AhR pathway) and IDO1 were both found to promote MDSC and Treg trafficking in addition to direct T cell anergy [100,101]. Because PD-1/PD-L1 interaction was found to induce IDO1 expression in dendritic cells [102], large-scale investigations into combined IDO1 blockade plus ICI were initiated [103]. While initial results were promising [103], the phase III clinical trial of epacadostat plus pembrolizumab vs. pembrolizumab alone did not show improved survival outcomes [104]. Despite these results, efforts to identify additional targets within the immunosuppressive Trp-Kyn-AhR pathway are still ongoing [105].

### 4.3. Lipids

Lipid metabolism is another area of active research as fatty acids (FA) play a significant role in tumor and immune cell function. Cancer cells upregulate various enzymes to both synthesize FAs de novo for proliferative purposes as well as catabolize FAs from neighboring cells for enhanced TCA throughput [106]. At the center of lipid metabolism, inflammation, and immune function lies prostaglandin E2 (PGE2), a lipid derivative. Researchers have demonstrated that tumor-induced PGE2 leads to MDSC and CAF activation in breast cancer models, differentiation of monocytes into TAMs in cervical cancer, and can also lead to COX-2 mediated angiogenesis in pancreatic cancer [107,108,109,110]. Additionally, as described by Herber et al., tumors with increased lipid content contain dendritic cells with dysfunctional antigen-presenting capabilities, likely secondary to impaired MHC-I trafficking [111,112]. Furthermore, inhibition of acetyl-CoA carboxylase led to normalization of lipid content and consequently restored dendritic cell function in a murine lymphoma model [111]. Overall, higher lipid content in tumors has been associated with increased metastatic potential and resistance to chemotherapy [113,114]. Additional evidence now points to the immunosuppressive characteristics of altered lipid metabolism; targeting these dysregulated pathways may be a viable combinatorial approach with current immune based therapies. As an example, Colacios et al. found that dysregulated sphingolipid metabolism, via sphingosine kinase 1 (SK1) and sphingomyelin synthase 1 (SMS1), in melanoma cells was associated with increased immunosuppressive markers and a more aggressive phenotype [115,116]. SK1-silenced murine models led to enhanced response to PD-1 and CTLA-4 blockade, promoting SK1 as a “checkpoint lipid kinase” that could modulate the immune state of the TME [115].

### 4.4. Lactate

**Figure 1:**
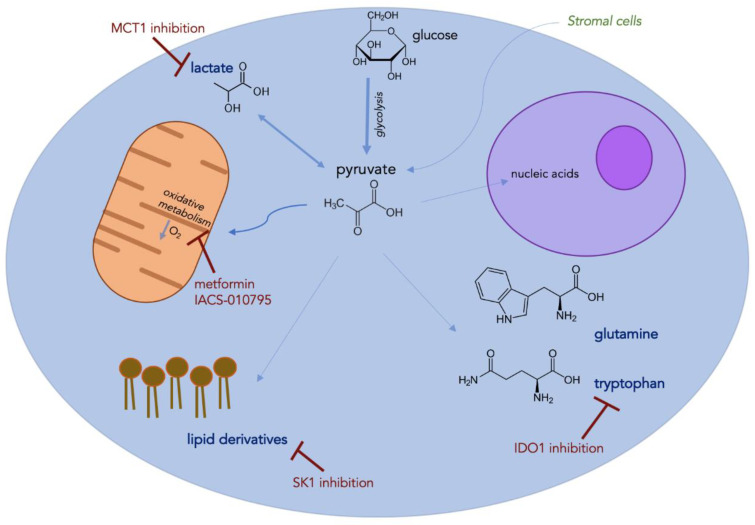
Tumor cell schematic highlighting known immunosuppressive metabolic factors with examples of proposed therapeutic interventions to be used alongside current immune-based therapies.

With enhanced glycolytic flux as described in such models as the reverse Warburg effect, increased lactate production leads to another important metabolic byproduct in the TME. While extracellular lactate can be shunted to normoxic tumor cells for enhanced proliferation, its effect on immune infiltration is more detrimental [94,117]. For example, increased lactate concentration can induce M2-polarization of TAMs as well as suppress NK function via enhanced MDSC trafficking in the TME [118,119]. Additionally, lactate has been shown to stimulate angiogenesis via a HIF-1-dependent process [120]. Peppicelli et al. studied the effects of lactate-mediated acidity in melanoma tumors; an increase in epithelial-to-mesenchymal transition was found (marker of aggressive phenotype) in addition to enhanced oxidative metabolism [121]. The OxPhos inhibitor, metformin, was applied to these tumors and resulted in a striking decrease in proliferative capacity in this acidic tumor subset [121]. Given the relevance of lactate flux throughout the TME, lactate transport (MCT) inhibitors have been proposed as a viable anti-cancer agent. A current MCT1 inhibitor under clinical investigation (AZD3965) has been shown to inhibit tumor growth via altered lipogenic enzyme activity; MCT1 blockade also led to enhanced dendritic and NK cell infiltration in this lymphoma xenograft model, highlighting the immunosuppressive effects of lactate [122]. Finally, an intriguing experiment by Benjamin et al. combined an MCT1/MCT4 inhibitor with metformin in a murine liver tumor model; this combined approach led to impaired NAD+ regeneration, a form of synthetic lethality secondary to intratumoral redox cessation [123]. Identifying efficacious drug regimens to combine with current IMT is certainly attainable (see Figure 1), however, this latter example highlights the more toxic ramifications that may preclude widespread applicability.

## 5. Conclusions

Checkpoint blockade and other immune-based therapies are now the standard of care for many cancers. However, given the relatively low and stagnant response rates seen with monotherapy, critical focus will be placed on improving the efficacy of these drugs over the next decade, by overcoming both primary and secondary resistance. While heterogenous and complex, characterizing the immune state of the TME is a key factor in this endeavor, and it is now clear that tumor-mediated metabolic perturbations are a primary driver of immune dysfunction. Hypoxia, oxidative stress, and other metabolic alterations can have a wide range of detrimental effects on T cell function, but further characterization also leads to important translational implications. Hypoxia, primarily through HIF-1-dependent processes, is strongly linked to an immunosuppressive phenotype via altered molecular markers, immune cell trafficking, and angiogenesis. Similarly, lipid peroxidation, ER stress, and Treg function can lead to immune dysregulation secondary to oxidative stress. Finally, a host of metabolic byproducts related to lipids, amino acids, glucose, and cellular energetics have been linked to T-cell suppression and ICI resistance. Targeting these alterations may help reverse the metabolic insufficiency of the TME and provide promising adjunctive therapies alongside current IMT.

## Figures and Tables

**Table 1:** Hypoxia-targeted drugs under investigation with select clinical trials.

Drug	Mechanism	Clinical Investigation
Evofosfamide (TH-302)	Pro-drug, converting to alkylating cytotoxic agent in hypoxic conditions	Phase I, evofosfamide plus ipilimumab in advanced solid cancers (NCT03098160) Phase I/II, evofosfamide plus bortezomib in relapsed/refractory MM (NCT01522872)
Girentuximab	Anti-CAIX antibody (upregulated via HIF-1)	Phase III, girentuximab vs. placebo in localized high-risk ccRCC (NCT00087022)
Ciforadenant (CPI-444)	Selective inhibitor of adenosine A2A receptor (A2AR)	Phase I/Ib, ciforadenant plus atezolizumab in advanced solid tumors (NCT02655822) Phase I/Ib, anti-CD73 plus ciforadenant plus pembrolizumab in advanced solid tumors (NCT03454451)
2ME2	Multifactorial inhibitor of HIF-1α and angiogenesis	Phase I, 2ME2 in advanced solid tumors (NCT00028821) Phase II, 2ME2 plus sunitinib in metastatic RCC (NCT00444314)
17-AAG	Inhibitor of HSP-90, increases degradation of HIF-1α	Phase II, 17-AAG in advanced melanoma (NCT00087386) Phase I, 17-AAG plus sorafenib in advanced solid tumors (NCT00121264)
Vorinostat	Inhibitor of histone deacetylase (HDAC), leads to HIF-1α degradation	Phase II, vorinostat plus pembrolizumab in recurrent HNSCC (NCT02538510)
PT2977	HIF-2α inhibitor (second generation)	Phase II, PT2977 plus cabozantinib in advanced RCC (NCT03634540)
EZN-2208	Metabolite of irinotecan, suppresses HIF-1 mRNA expression	Phase II, EZN-2208 plus cetuximab in metastatic CRC (NCT00931840)
CRLX101	Nanoparticle conjugate with camptothecin, inhibits HIF-1α	Phase II, CRLX101 plus bevacizumab in smetastatic RCC (NCT02187302)
